# A non-invasive method to generate induced pluripotent stem cells from primate urine

**DOI:** 10.1038/s41598-021-82883-0

**Published:** 2021-02-10

**Authors:** Johanna Geuder, Lucas E. Wange, Aleksandar Janjic, Jessica Radmer, Philipp Janssen, Johannes W. Bagnoli, Stefan Müller, Artur Kaul, Mari Ohnuki, Wolfgang Enard

**Affiliations:** 1 Anthropology and Human Genomics, Department of Biology II, Ludwig-Maximilians-University, Großhaderner Straße 2, 82152 Martinsried, Germany; 2 Institute of Human Genetics, Munich University Hospital, Ludwig-Maximilians-University Munich, 80336 Munich, Germany; 3 Infection Biology Unit, German Primate Center, 37077 Göttingen, Germany

**Keywords:** Evolution, Cell biology

## Abstract

Comparing the molecular and cellular properties among primates is crucial to better understand human evolution and biology. However, it is difficult or ethically impossible to collect matched tissues from many primates, especially during development. An alternative is to model different cell types and their development using induced pluripotent stem cells (iPSCs). These can be generated from many tissue sources, but non-invasive sampling would decisively broaden the spectrum of non-human primates that can be investigated. Here, we report the generation of primate iPSCs from urine samples. We first validate and optimize the procedure using human urine samples and show that suspension- Sendai Virus transduction of reprogramming factors into urinary cells efficiently generates integration-free iPSCs, which maintain their pluripotency under feeder-free culture conditions. We demonstrate that this method is also applicable to gorilla and orangutan urinary cells isolated from a non-sterile zoo floor. We characterize the urinary cells, iPSCs and derived neural progenitor cells using karyotyping, immunohistochemistry, differentiation assays and RNA-sequencing. We show that the urine-derived human iPSCs are indistinguishable from well characterized PBMC-derived human iPSCs and that the gorilla and orangutan iPSCs are well comparable to the human iPSCs. In summary, this study introduces a novel and efficient approach to non-invasively generate iPSCs from primate urine. This will extend the zoo of species available for a comparative approach to molecular and cellular phenotypes.

## Introduction

Primates are our closest relatives and hence play an essential role in comparative and evolutionary studies in biology, ecology and medicine. We share the vast majority of our genetic information, and yet have considerable molecular and phenotypic differences^1^. Understanding this genotype–phenotype evolution is crucial to understand the molecular basis of human-specific traits. Additionally, it is biomedically highly relevant to interpret findings made in model organisms, such as the mouse, and to identify the conservation and functional relevance of molecular and cellular circuitries^2,3^. However, obtaining comparable samples from different primates, especially during development, is practically and—more importantly—ethically very difficult or even impossible.

Embryonic stem cells have the potential to partially overcome this limitation by their ability to differentiate into all cell types in vitro and divide indefinitely^4^. However, the necessary primary material collection from an embryo is in most cases impossible. Fortunately, a pluripotent state can also be induced in somatic cells by ectopically expressing four genes^5^. Since this discovery of induced pluripotency, great efforts have been made to identify suitable somatic cells^6^ and optimize reprogramming methods^7^. Most of this research, however, has focused on human or mouse. While the methods are generally transferable and iPSCs from several different non-human primates^8–10^ and other mammals^11,12^ have been generated, these methods have not been optimized for non-model organisms.

One major challenge for establishing iPSCs of various non-human primates is the acquisition of the primary cells. So far iPSCs have been generated from fibroblasts, peripheral blood cells or vein endothelial cells derived during medical examinations or from post mortem tissue^8–10,13,14^. However, also these sources impose practical and ethical constraints and therefore limit the availability of the primary material.

**Figure 1:**
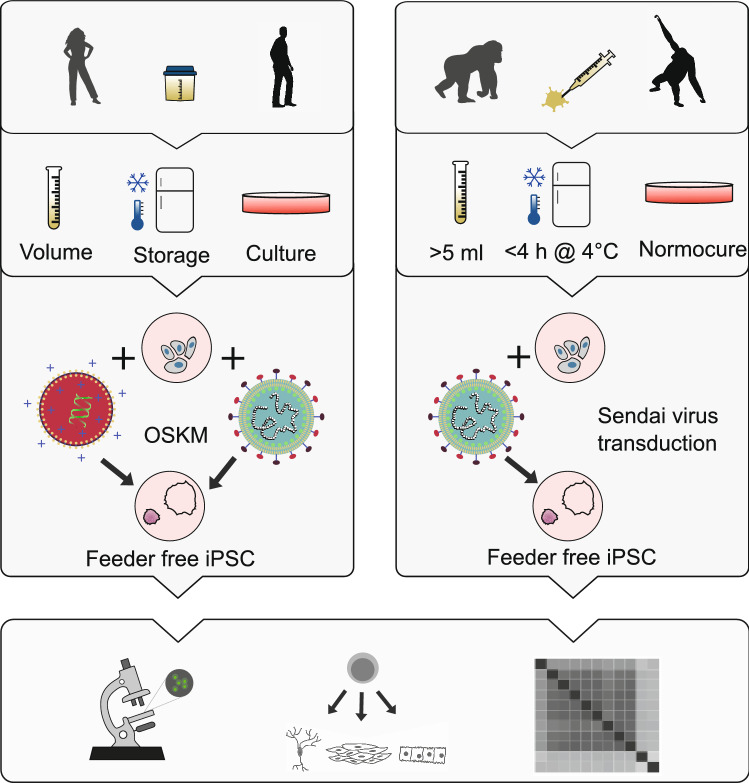
Workflow overview for establishing iPSCs from primate urine. We established the protocol for iPSC generation from human urine based on a previously described protocol^16^. We tested volume, storage and culture conditions for primary cells and compared reprogramming by overexpression of OCT3/4, SOX2, KLF4 and MYC (OSKM) via lipofection of episomal vectors and via transduction of a Sendai virus derived vector (SeV). We used the protocol established in humans and adapted it for unsterile floor-collected samples from non-human primates by adding Normocure to the first passages of primary cell culture and reprogrammed visually healthy and uncontaminated cultures using SeV. Pluripotency of established cultures was verified by marker expression, differentiation capacity and cell type classification using RNA sequencing.

To overcome these limitations, we adapted a method of isolating reprogrammable cells from human urine samples^15,16^ and applied it to non-human primates (Fig. 1). We find that primary cells can be isolated from unsterile urine sampled from the floor, can be efficiently reprogrammed using the integration-free Sendai Virus^17^ and can be maintained under feeder-free conditions as shown by generating iPSCs from human, gorilla and orangutan.

## Results

### Isolating human urinary cells from small-volume and stored samples

**Figure 2:**
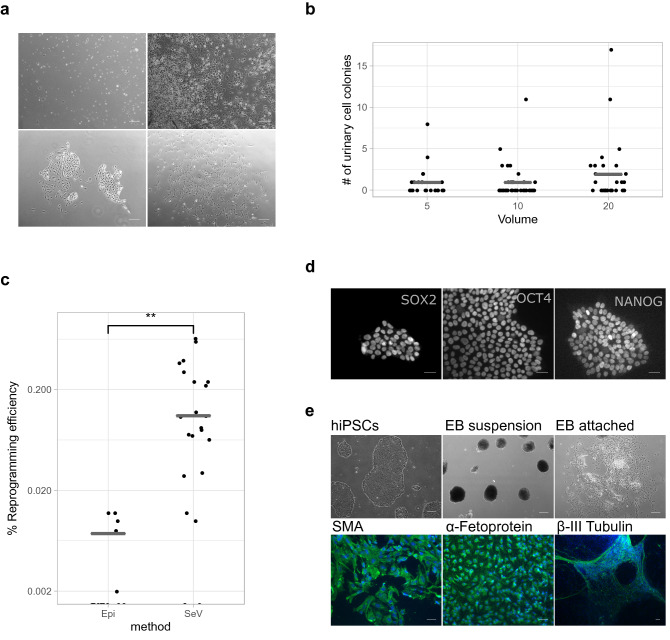
Establishing urinary cell isolation and reprogramming to iPSCs in human samples. (**a**) Human urine mainly consists of squamous cells and other differentiated cells that are not able to attach and proliferate (upper row). After ~ 5 days, the first colonies become visible and two types of colonies can be distinguished as described in Zhou (2012). Scale bars represent 500 μm. (**b**) Isolation efficiency of urine varies between samples. The efficiency between 5 ml, 10 ml and 20 ml of starting material is not different (Fisher’s exact test *p* > 0.5). (**c**) SeV mediated reprogramming showed significantly higher efficiency than Episomal plasmids (Wilcoxon rank sum test: p = 1.1e−05). (**d**) Established human colonies transduced with SeV expressed Nanog, Oct4 and Sox2; Scale bars represent 50 μm and (**e**) differentiated to cell types of the three germ layers; scale bar represents 500 µm in the phase contrast pictures and 100 µm in the fluorescence pictures. See also Supplementary Figure S1.

To assess which method is most suitable for isolating and reprogramming primate cells, we first tested different procedures using urinary cells from human samples (Fig. 1). We collected urine from several humans in sterile beakers and processed them as described in Zhou et al.^15,16^. We found varying cell numbers in the urine samples (range 46–2250 cells per ml; Supplementary Table S1) with about 60% living cells. As previously reported^18,19^, we initially observed two morphologically distinct colony types that became indistinguishable after the first passage and consisted of grain-shaped cells that proliferated extensively (Fig. 2a, Supplementary Figure S1b). In total we processed 19 samples of several individuals in 122 experiments using different volumes and storage times (Supplementary Table S2). Similar to previous reports^20^, we isolated an average of 7.6 colonies per 100 ml of urine when processing samples immediately with a considerable amount of variation among samples (0–70 colonies per 100 ml, Supplementary Table S2) and among aliquots (0–160 per 100 ml; Supplementary Table S2; Fig. 2b), but no difference between sexes (Supplementary Table S2). Furthermore, storing samples for up to 4 h at room temperature or on ice did not influence the number of isolated colonies (9 samples, 7.4 colonies on average per 100 ml, range: 0–17). As sample volumes can be small for non-human primates, we also tested whether colonies can be isolated from 5, 10 or 20 ml of urine (Fig. 2b). We found no evidence that smaller volumes have lower success rates as we found that for 42% of the 5 ml samples, we could isolate at least one colony (Supplementary Table S2). Many more samples and conditions would be needed to better quantify the influence of different parameters on the isolation efficiency of colonies. However, in most practical situations such parameters would not be used to make a decision as one would anyway try to obtain colonies with the urine samples at hand, especially in our case where samples from primates are rare. Fortunately, low-volume human urine samples stored for a few hours at room temperature or on ice are a possible source to establish primary urinary cell lines. In summary, these experiments are a promising starting point for the use of small-volume urine samples from non-human primates to generate primary cell lines, which may then be reprogrammed into iPSCs.

### Reprogramming human urinary cells is efficient when using suspension-Sendai Virus transduction

Next, we investigated which integration-free overexpression strategy would be the most suitable to induce pluripotency in the isolated urine cells. To this end we compared transduction by a vector derived from the RNA-based Sendai Virus^14,17^ in suspension^10^, to lipofection with episomal plasmids (Epi) derived from the Epstein Barr virus^21,22^. We chose to use the suspension transduction method as it yielded a significantly higher reprogramming efficiency than the method on attached cells (suspension reprogramming efficiency: 0.24%, N = 7; attached reprogramming efficiency: 0.09%, N = 7; Wilcoxon rank sum test: p = 0.003; Supplementary Table S3, Supplementary Figure S2d). Both systems have been previously reported to sufficiently induce reprogramming of somatic cells without the risk of genome integrations. In our experiments presented here, transduction of urinary cells with a Sendai Virus (SeV) vector containing Emerald GFP (EmGFP) showed substantially higher efficiencies than lipofection with episomal plasmids (~ 97% versus ~ 20% EmGFP+; Supplementary Figure S2a and S2b). We assessed the reprogramming efficiency of these two systems by counting colonies with a pluripotent-like cell morphology. Using SeV vectors, 0.19% of the cells gave rise to such colonies (Fig. 2c). In contrast, when using Episomal plasmids only 0.009% of the cells gave rise to colonies with pluripotent cell-like morphology (N = 23 and 18, respectively; Wilcoxon rank sum test: p = 0.00005), resulting in at least one colony in 87% and 28% of the cases. Furthermore, the first colonies with a pluripotent morphology appeared 5 days after SeV transduction and 14 days after Epi lipofection. To test whether the morphologically defined pluripotent colonies also express molecular markers of pluripotency, we isolated flat, clear-edged colonies from 5 independently transduced urinary cell cultures on day 10. All clones expressed POU5F1 (OCT3/4), SOX2, NANOG and differentiated into the three germ layers during embryoid body formation as shown by immunocytochemistry (Fig. 2d,e). Notably, while the transduced cells also expressed the pluripotency marker SSEA4, this was also true for the primary urinary cells (Supplementary Figure S2c). SSEA4 is known to be expressed in urine derived cells^18,23^ and hence it is an uninformative marker to assess the reprogramming of urinary cells to iPSCs. Furthermore, SeV RNA was always absent after the first five passages (Supplementary Figure S3) and the pluripotent state could be maintained for over 100 passages (data not shown).

In summary, we find that the generation of iPSCs from human urine samples is possible from small volumes, and our results also reveal that reprogramming is most efficient when using suspension SeV transduction. Hence, we used this workflow for generating iPSCs from non-human primate cells.

### Isolating cells from unsterile primate urine

For practical and ethical reasons, the collection procedure is a decisive difference when sampling urine from non-human primates (NHPs). Samples from chimpanzees, gorillas and orangutans were collected by zoo keepers directly from the floor, often with visible contamination. Initially, culturing these samples was not successful due to the growth of contaminating bacteria. The isolation and culture of urinary cells only became possible upon the addition of Normocure (Invivogen), a broad-spectrum antibacterial agent that actively eliminates Gram+ and Gram− bacteria from cell cultures. We confirmed that Normocure did not affect the number of colonies isolated from sterile human samples (Supplementary Table S2). Furthermore, many NHP samples also had volumes below 5 ml. We attempted to isolate cells from a total of 70 samples, but only 24 NHP samples showed collection parameters comparable to human urine samples as described above (≥ 5 ml of sample, < 4 h storage at RT or 4 °C and no visible contamination). From chimpanzees, gorillas and orangutans we collected a total of 87, 70 and 39 ml of urine in 11, 8 and 5 samples from several individuals and isolated 0, 5 and 2 colonies respectively (Supplementary Table S4). For gorilla and orangutan this rate (7.3 and 5.2 colonies per 100 ml urine) is not significantly different from the rate found for human samples (6.0 per 100 ml across all conditions in Supplementary Table S2, p = 0.8 and 0.6, respectively, assuming a Poisson distribution). However, obtaining zero colonies from 87 ml of chimpanzee urine is less than expected, given the rate found in human samples (p = 0.005). While isolating primary cells from urine samples seems comparable to humans in two great ape species, it seems to have at least a two- to threefold lower rate in our closest relatives, suggesting that the procedure might work in many but not in all NHPs. Fortunately, it is possible to culture many samples in parallel so that screening for urinary cells in a larger volume with more samples is relatively easy.

**Figure 3:**
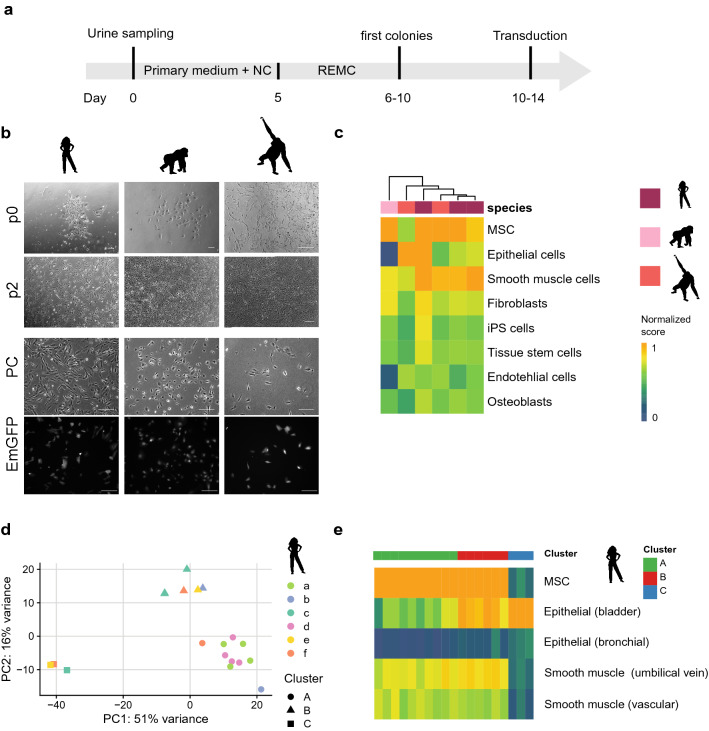
Isolation and characterization of primate urinary cells. (**a**) Workflow of cell isolation from primate urine samples. *NC *Normocure, *REMC *renal epithelial mesenchymal cell medium. (**b**) Primary cells obtained from human, gorilla and orangutan samples are morphologically indistinguishable and display similar EmGFP transduction levels. Scale bars represent 400 μm. (**c**) The package SingleR was used to correlate the expression profiles from six samples of primate urinary cells (passage 1–3) to a reference set of 38 human cell types. Normalized scores of the eight cell types with the highest correlations are shown (*MSC *mesenchymal stem cells, *SM *smooth muscle, *Epi *epithelial, *Endo *endothelial). Color bar indicates normalized correlation score. (**d**) Principal component analysis of primary cells from single colony lysates using the 500 most variable genes. (**e**) Heatmap of normalized SingleR scores show that cluster C is classified as epithelial cell originating from the bladder. The scores for MSCs in Cluster A and B are similarly high, although cluster B also shows higher scores for epithelial cells than cluster A. See also Supplementary Figure S5.

The first proliferating cells from orangutan and gorilla could be observed after six to ten days (Fig. 3a,b) in culture and could be propagated for several passages, which is comparable to human cells. While we observed different proliferation rates and morphologies among samples, these did not systematically differ among individuals or species (Fig. 3b). Infection with specific pathogens, including simian immunodeficiency virus (SIV), herpes B virus (BV, Macacine alphaherpesvirus 1), simian T cell leukemia virus (STLV) and simian type D retroviruses (SRV/D), was not detected in these cells (data not shown).

### Expression patterns of urinary cells are most similar to mesenchymal stem cells, epithelial cells and smooth muscle cells

To characterize the isolated urinary cells, we generated expression profiles using prime-seq a 3′ tagged RNA-seq protocol^24–26^, on early passage primary urinary cells (p1–3) from three humans, one gorilla and one orangutan. Note that some of these samples contained cells from 1–4 different colonies (Supplementary Table S2 and S4) and hence could be mixtures of different cell types. To classify these urinary cells we compared their expression profile to 713 microarray expression profiles grouped into 38 cell types^27^ using the SingleR package^28^. SingleR uses the most informative genes from the reference dataset and iteratively correlates it with the expression profile to be classified. The most similar cell types were mesenchymal stem cells, epithelial cells and/or smooth muscle cells and at least two groups are evident among the six samples (Fig. 3c). To further investigate these cell types, we isolated 19 single colonies from six different individuals (Supplementary Table S1) and analyzed their expression profiles as described above. A principal component analysis revealed three clearly distinct clusters A, B and C with 10, 6 and 3 colonies, respectively (Fig. 3d). When we classified these 19 profiles using SingleR^27,28^ as described above, we found the three colonies from cluster C clearly classified as epithelial cells from the bladder (Fig. 3e). This cluster shows high KRT7 expression, as also described in Dörrrenhaus et al.^19^ as well as high FOXA1 expression, both hinting towards an urothelial origin (Supplementary Figure S4). The colonies of the other two clusters are classified as MSCs, whereas cluster B also has a high similarity to epithelial profiles (Fig. 3e). They could resemble the two renal cell types described in Dörrrenhaus et al.^19^ and are probably derived from the kidney as also evident by their PAX2 and MCAM expression (Supplementary Figure S4). We also used differential gene expression and Reactome pathway analysis^29^ to further characterize the differences between these clusters (Supplementary Figure S4a, S4c). In sum, our findings indicate that at least three types of proliferating cells can be isolated from urine, one of urothelial and two of renal origin and that the same types can also be isolated from gorilla and orangutan.

### Reprogramming efficiency of urinary cells is similar in humans and other primates

**Figure 4:**
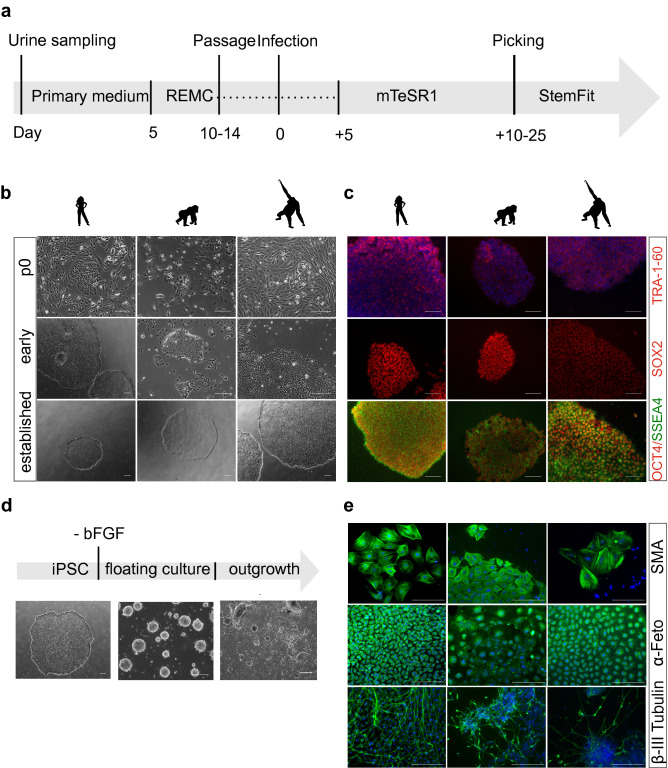
Generation and characterization of primate iPSCs. (**a**) Workflow for reprogramming of primate urinary cells. Urine collection and cell seeding is carried out in primary medium, then after 5 days changed to REMC medium, and only passaged for the first time after 10–14 days. When the cells reach confluency reprogramming is induced and after 5 days the medium is changed to mTeSR1. Once the reprogrammed cells are ready to be picked, the cells are seeded in StemFit medium. *REMC *renal epithelial mesenchymal cell medium. (**b**) Cell morphology of the three species is comparable before (p0), during (p1–3) and after reprogramming (~ p5). Scale bar represents 400 µm. (**c**) Immunofluorescence analysis of pluripotency associated proteins at passage 10–15: TRA-1-60, SSEA4, OCT4 and SOX2. Nuclei were counterstained with DAPI. Scale bars represent 200 µm. (**d**) Differentiation potency into the three germ layers. iPSC colony before differentiation, after 8 days of floating culture and after 8 days of attached culture. Scale bar represents 400 µm. (**e**) Immunofluorescence analyses of ectoderm (β-III Tubulin), mesoderm (α-SMA) and endoderm markers (α-Feto) after EB outgrowth. Nuclei were counterstained with DAPI. Scale bars represent 400 μm. See also Supplementary Figure S7a.

To generate iPSCs from the urinary cells isolated from gorilla and orangutan, we used Sendai Virus (SeV) transduction and the reprogramming timeline that we found to be efficient for human urinary cells (Fig. 4a). Human, gorilla and orangutan urinary cells showed similarly high transduction efficiencies with the EmGFP SeV vector (data not shown). Transduction with the reprogramming SeV vectors led to initial morphological changes after 2 days in all three species, when cells began to form colonies and became clearly distinguishable from the primary cells (Fig. 4b). When flat, clear-edged colonies appeared that contained cells with a large nucleus to cytoplasm ratio, these colonies were picked and plated onto a new dish. We found that the efficiency and speed of reprogramming was variable (Supplementary Figure S5b), probably depending on the cell type, the passage number and the acute state (“health”) of the cells, in concordance with the variability and efficiency found in other studies utilizing urine cells as a source for iPSCs^15^. Also the mean reprogramming efficiency over all replicates was different (Kruskal–Wallis test, p = 0.015) for human (0.19%), gorilla (0.28%) and orangutan (0.061%). However, many more samples would be necessary to disentangle the effects of all these contributing factors. Of note, we observed that the orangutan iPSCs showed more variability in proliferation rates and morphology compared to human and gorilla iPSCs. Several subcloning steps were needed until a morphologically stable clone could be generated. However, the resulting iPSCs were stable and had the same properties as the other iPSCs (Fig. 4). To what extent this is indeed a property of the species is currently unclear. Importantly, from all primary samples that were transduced, colonies with an iPSC morphology could be obtained. So, while considerable variability in reprogramming efficiency exists, the overall success rate is sufficiently high and sufficiently similar in humans, gorillas and orangutans.

### Urine derived primate iPSCs are comparable to human iPSCs

We could generate at least two lines per individual from each primary cell sample, all of which showed Oct3/4, TRA-1-60, SSEA4 and SOX2 immunofluorescence (Fig. 4c). Furthermore, karyotype analysis by G-banding in three humans, one gorilla and one orangutan iPS cell line revealed no recurrent numerical or structural aberrations in 33–60 metaphases analyzed per cell line. All five cell lines analyzed showed inconspicuous and stable karyotypes (Supplementary Figure S6). iPSCs from all species could be expanded for more than fifty passages, while maintaining their pluripotency, as shown by pluripotency marker expression (Fig. 4c) and differentiation capacity via embryoid body formation (Fig. 4d,e). Both the human and NHP iPSCs differentiated into ectoderm (beta-III Tubulin), mesoderm (α-SMA) and endoderm (AFP) lineages (Fig. 4e, Figure S7a). Dual-SMAD inhibition led to the formation of neurospheres in floating culture, as confirmed by neural stem cell marker expression (NESTIN+, PAX6+) using qRT-PCR (Supplementary Figure S7b).

To further assess and compare the urine-derived iPSCs, we generated RNA-seq profiles from nine human, three gorilla and four orangutan iPSC lines as well as the six corresponding primary urinary cells (see analysis above). As an external reference, we added a previously reported and well characterized blood-derived human iPS cell line that was generated using episomal vectors and adapted to the same feeder-free culture conditions as our cells (1383D2)^30^. All lines were grown and processed under the same conditions and in a randomized order in one experimental batch. We picked one colony per sample and used prime-seq, a 3′ tagged RNA-seq protocol^24–26^ to generate expression profiles with 19,000 genes detected on average.

**Figure 5:**
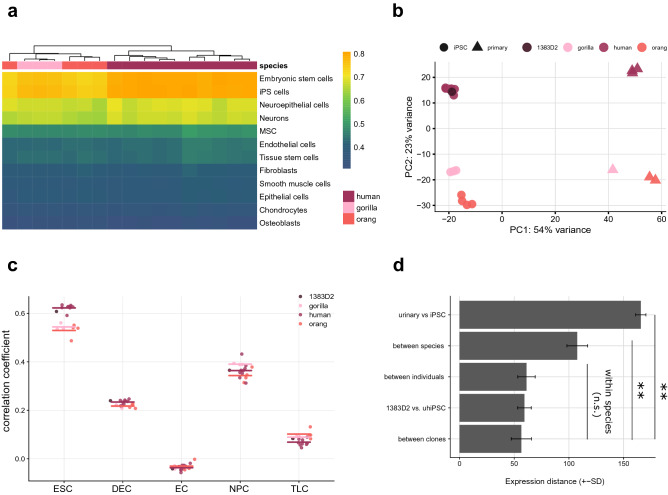
Characterization of primate iPSCs by expression profiling. (**a**) The package SingleR was used to correlate the expression profiles from seventeen samples of primate iPSCs (passage 1–3) to a reference set of 38 human cell types. The twelve cell types with the highest correlations are shown (*MSC *mesenchymal stem cells). All lines are similarly correlated to embryonic stem cells and iPS cells. Color bar indicates correlation coefficients. (**b**) Principal component analysis of primary cells and derived iPSC lines using the 500 most variable genes. PC1 separates the cell types and PC2 separates the species from each other. (**c**) Correlation coefficient of iPSCs compared to a single cell dataset covering distinct human embryonic stem cell derived progenitor states (Chu et al. 2016). (**d**) Expression distances of all detected genes are averaged from pairwise distances for six different groups of comparisons. Note that the distance between individuals and between species is calculated within iPSCs and distances between individuals within species. Pairwise t-tests are all below 0.01 (**) for comparisons to the cell-type and species distance and all above 0.05 (n.s.) for comparisons within the species. See also Supplementary Figure S5.

We classified the expression pattern of the iPSCs relative to the reference dataset of 38 cell types using SingleR as described for the urinary cells. ES cells or iPS cells are clearly the most similar cell type for all our iPS samples including the external PBMC-derived iPSC line (Fig. 5a). Principal component analysis of the 500 most variable genes (Fig. 5b), shows clear clustering of the samples according to cell type (54% of the variation in PC1) and species (23% of the variance in PC2). The external, human blood-derived iPSC line is interspersed among our human urine derived iPS cell lines. Using the pairwise Euclidean distances between samples to assess similarity, they also cluster first by cell type and then by species (Supplementary Figure S5d). When classifying the expression pattern of the iPSCs relative to a single cell RNA-seq dataset covering distinct human embryonic stem cell derived progenitor states (Chu et. al. 2016), again all our iPSC lines are most similar to embryonic stem cells and are indistinguishable from the external PBMC-derived iPSC line (Fig. 5c), also confirming the immunostainings. Finally, expression distances within iPS cells of the same species were similar, independent of the individual and donor cell type (Fig. 5d).

Taken together, these analyses do not only indicate that our urine derived iPS cells show a pluripotent expression profile and differentiate as expected for iPS cells but can also not be distinguished from an iPSC line derived in another laboratory from another cell type with another vector system. Hence, the expression differences among species are far larger than these technical sources of variation, indicating that these cells are well suited to assess species differences among primates in iPS cells as well as in cell types derived from these pluripotent cells by in vitro differentiation strategies.

## Discussion

Here, we adapted a previously described protocol for human urine samples^16^ to isolate proliferating cells from unsterile primate urine. We show that these urinary cells can be efficiently reprogrammed into integration-free and feeder-free iPSCs, which are closely comparable among each other and to other iPSCs. Our findings have implications for generating and validating iPSCs from primates and other species for comparative studies. Additionally, some aspects might also be of relevance when generating iPSCs from human urinary cells for medical studies.

Human urine mainly contains cells, such as squamous cells, which are terminally differentiated and cannot attach or proliferate in culture. The first proliferating cells from human urine were isolated in 1972^31^ and since then a variety of different cells have been isolated and described that can proliferate, differentiate and be reprogrammed to iPSCs (see^32^ for a recent overview). As these urine-derived stem cells (UDSCs) can be isolated non-invasively at low costs and reprogrammed efficiently^16^, they are increasingly used to generate iPSCs from patients (e.g.^33–35^). Perhaps the only major drawback of using UDSCs for iPSC generation is that the number of UDSCs that can be grown per milliliter is quite variable among samples. While parameters such as body size, age and cell count correlate with the number of isolated colonies^20^, isolation can fail despite large volumes and can be successful despite small volumes (Supplementary Table S1, Supplementary Table S2). As UDSC culturing is neither very cost- nor time-intensive, the best practical solution will in most cases be to try isolating UDSCs independent of those parameters.

While it is known for a long time that different types of UDSCs can be isolated, the quantitative relation between morphology, marker expression, potency and reprogramming efficiency among the different UDSCs is not clear. The RNA-seq profiles of single colonies presented here, allow for the first time to classify them based on genome-wide expression patterns. In agreement with previous findings using marker staining and morphological analysis^19^, we find three different cell types, of which one is most similar to epithelial cells from the bladder and the other two are most similar to mesenchymal stem cells and probably originate from the kidney. Importantly, all three cell types seem to reprogram with sufficient efficiency and the expression of pluripotency markers like KLF4 and OCT3/4 in all three cell types (Supplementary Figure S4) might be one factor why the reprogramming efficiency of UDSCs is relatively high compared to other primary cells. Regarding the reprogramming method, we find that transduction using the commercial Sendai Virus based vector in suspension^10^ is substantially more efficient for UDSCs than lipofection of episomal plasmids, and also leads to a change in morphology within 2 days. While it is established that Sendai Virus reprogramming is an expensive but efficient method to generate iPSCs from fibroblasts^7,36^, our findings indicate that the suspension method might be especially efficient for UDSCs. Finally, a relevant side note of our findings is that SSEA4, which is occasionally used as a marker for pluripotency^37,38^, is not useful when starting from urinary cells as these express SSEA4 at already high levels (Supplementary Figure S2c). In summary, our findings contribute to a better understanding of human UDSCs and to a method to more efficiently reprogram them into iPSCs.

Maybe more important are the implications of our study for isolating urinary stem cells for the generation of iPSC from primates and other mammals. This could be useful in contexts where invasive sampling is difficult, as it is the case for non-model primates and many other mammals, and where iPSCs are needed for conservation^11^ or comparative approaches as discussed below. So how likely is it that one can find UDSCs in other primates and mammals? In humans, UDSCs originate from the kidney and the urinary tract as also shown by our transcriptional profiles. We isolated UDSCs from orangutan and gorilla and found similar transcriptional profiles, morphologies and growth characteristics. Given the general similarity of the urinary tract in mammals and our successful isolation of UDSCs in two apes, it seems likely that most primates, and maybe even most mammals, shed UDSCs in their urine. However, our failure to isolate UDSCs from chimpanzees suggests that even very closely related species might have at least 2–3 times less of those cells in their urine. An alternative possibility is that the culture conditions, e.g. the FBS, do not work for isolating chimpanzee UDSCs. However, given that UDSCs from gorilla and orangutan can be isolated under these conditions and fetal calf serum works for tissue cultures of chimpanzee kidneys^39^, we think that a lower concentration of UDSCs in some species is the more likely cause. Hence, from which species UDSCs can be isolated in practice might depend mainly on the concentration of UDSCs and the available amount of urine. Fortunately, this can be easily tested for any given species of interest, as culturing systems are very cost-efficient. Furthermore, our procedure to use unsterile samples from the ground to isolate such cells broadens the practical implementation of this approach considerably.

Given that it is possible to isolate UDSCs from a species, the efficiency of reprogramming and iPSC maintenance will determine whether one can generate stable iPSCs from them. Fortunately, the efficiency of reprogramming UDSCs is shown to be high, probably higher than for many other primary cell types^6^. This is especially true when using SeV transduction in suspension as is evident from the fact that we could generate iPSCs from all twelve UDSC reprogramming experiments (Supplementary Table S5). To what extent this reprogramming procedure works in other species is currently unclear, but as the Sendai virus is thought to infect all mammalian cells^40^ it could be widely applicable. Additionally, iPSCs have been previously generated from many species, even avian species^11^, when using human reprogramming factors and culture conditions, albeit with over tenfold lower reprogramming efficiencies^41,42^. So, while in principle it should be possible to isolate iPSCs from many or even all mammals, variation in reprogramming efficiency with human factors and culture conditions to keep cells pluripotent with and without feeder cells^42^ will considerably vary among species and will make it practically difficult to obtain and maintain iPSCs from some species. Investigating the cause of this variation more systematically will be important to better understand pluripotent stem cells in general and to generate iPSCs from many species in practice. Recent examples of such fruitful investigations include the optimization of culture conditions for baboons^43^, and the optimization of feeder-free culture conditions for rhesus macaques and baboons^42^. A related aspect of generating iPSCs from different species is testing whether iPSCs from a given species are actually *bona fide* iPSCs. While for humans a variety of tools exist, such as predictive gene expression assays, validated antibody stainings and SNP arrays for chromosomal integrity, these tools cannot be directly transferred to other species. Fortunately, due to the availability of genome sequences, RNA-sequencing in combination with human or mouse reference cell types to which generated iPSCs can be compared, but also rather traditional techniques such as karyotyping, the characterization of non-human iPSCs becomes feasible as also shown in this paper. In summary, while extending the zoo of comparable iPSCs is a daunting task and requires considerable more method development, we think our method to isolate UDSCs from unsterile urine could be a promising tool in this endeavor.

Assuming that our approach works in at least some non-human primates (NHPs), the effectiveness and non-invasiveness of the protocol allows sampling many more individuals and species than currently possible. Why is this important? So far, iPSCs have been generated from only a few individuals in a very limited set of NHP species. One main application is to model biomedical applications of iPSCs in primates such as rhesus macaques or marmosets^44^. As these species are used as model organisms, non-invasive sampling is less of an issue. Another main application are studies investigating the molecular basis of human-specific phenotypes e.g. by comparing gene expression levels in humans, chimpanzees and an outgroup^8,9,45,46^ to infer human-specific changes more robustly^47^. A third type of application with considerable potential has been explored much less, namely using iPSCs in a comparative framework to identify molecular or cellular properties that are conserved, i.e. functional across species^2,3,48^. This is similar to the comparative approach on the genotype level in which DNA or protein sequences are compared in orthologous regions among several species to identify conserved, i.e. functional elements^49^. This information is crucial, for example, when inferring the pathogenicity of genetic variants^50^. Accordingly, it would be useful to know whether a particular phenotypic variant, e.g. a disease associated gene expression pattern, is conserved across species. This requires a comparison of the orthologous cell types and states among several species. Primates are well suited for such an approach, because they bridge the evolutionary gap between human and its most important model organism, the mouse, and because phenotypes and orthologous cell states can be more reliably compared in closely related species. However, for practical and ethical reasons, orthologous cell states are difficult to obtain from several different primates. Hence, just as human iPSCs allow one to study cell types and states that are for practical and ethical reasons not accessible, primate iPSCs extend the comparative approach to these cell types and states, leveraging unique evolutionary information that is not only interesting per se, but could also be of biomedical relevance. As our method considerably extends the possibilities to derive iPSCs from primates, it could contribute towards leveraging the unique information generated during millions of years of primate evolution.

## Methods

### Experimental model and subject details

#### Human urine samples

Human urine samples from healthy volunteers were obtained with written informed consent and processed anonymously. This experimental procedure was ethically approved by the responsible committee on human experimentation (20-122, Ethikkommission LMU München). All experimental procedures were performed in accordance with relevant guidelines and regulations. Additional information on the samples is available in Supplementary Table S2.

#### Primate urine samples

Primate urine was collected at the Hellabrunn Zoo in Munich, Germany. Caretakers noted the time and most likely donor and took up available urine on the floor with a syringe, hence the collection procedure was fully non-invasive without any perturbation of the animals. Due to the collection procedure we do not know with certainty from which individual the samples were derived. Additional information on the samples can be found in Supplementary Table S4.

#### iPSC lines

iPSC lines were generated from human and non-human primate urinary cells. Reprogramming was done using two different techniques. Reprogramming using SeV (Thermo Fisher) was performed as suspension transduction as described before^10^. Episomal vectors were transfected using Lipofectamine 3000 (Thermo Fisher). iPSCs were cultured under feeder-free conditions on Geltrex (Thermo Fisher) -coated dishes in StemFit medium (Ajinomoto) supplemented with 100 ng/ml recombinant human basic FGF (Peprotech), 100 U/ml Penicillin and 100 μg/ml Streptomycin (Thermo Fisher) at 37 °C with 5% carbon dioxide. Cells were routinely subcultured using 0.5 mM EDTA. Whenever cells were dissociated into single cells using 0.5 × TrypLE Select (Thermo Fisher) or Accumax (Sigma Aldrich), the culture medium was supplemented with 10 µM Rho-associated kinase (ROCK) inhibitor Y27632 (BIOZOL) to prevent apoptosis.

### Isolation of cells from urine samples

Urine from human volunteers was collected anonymously in sterile tubes. Usually a volume of 5–50 ml was obtained. Urine from NHPs was collected from the floor at Hellabrunn Zoo (Munich) by the zoo personnel, using a syringe without taking special precautions while collecting the samples. Samples were stored at 4 °C until processing for a maximum time span of 5 h. Isolation of primary cells was performed as previously described by Zhou et al. 2012. Briefly, the sample was centrifuged at 400×*g* for 10 min and washed with DPBS containing 100 U/ml Penicillin, 100 μg/ml Streptomycin (Thermo Fisher), 2.5 µg/ml Amphotericin (Sigma-Aldrich). Afterwards, the cells were resuspended in urinary primary medium consisting of 10% FBS (Life Technologies), 100 U/ml Penicillin, 100 μg/ml Streptomycin (Thermo Fisher), REGM supplement (ATCC) in DMEM/F12 (TH. Geyer) and seeded onto one gelatine coated well of a 12-well-plate. To avoid contamination stemming from the unsanitary sample collection, 100 µg/ml Normocure (Invivogen) was added to the cultures until the first passage. 1 ml of medium was added every day until day 5, where 4 ml of the medium was aspirated and 1 ml of renal epithelial and mesenchymal cell proliferation medium RE/MC proliferation medium was added. RE/MC consists of a 50/50 mixture of Renal Epithelial Cell Basal Medium (ATCC) plus the Renal Epithelial Cell Growth Kit (ATCC) and mesenchymal cell medium consisting of DMEM high glucose with 10% FBS (Life Technologies), 2 mM GlutaMAX-I (Thermo Fisher), 1 × NEAA (Thermo Fisher), 100 U/ml Penicillin, 100 μg/ml Streptomycin (Thermo Fisher), 5 ng/ml bFGF (PeproTech), 5 ng/ml PDGF-AB (PeproTech) and 5 ng/ml EGF (Miltenyi Biotec). Half of the medium was changed every day until the first colonies appeared. Subsequent medium changes were performed every second day. Passaging was conducted using 0.5 × TrypLE Select (Thermo Fisher). Typically 15 × 10^3^ to 30 × 10^3^ cells were seeded per well of a 12-well plate.

### Single colony isolation from urine samples

For the UDSC single colony characterization experiment we seeded cells of 3 ml urine sample per well and chose the wells with only one colony for further characterization. The cells grew without further passage for two weeks (some colonies appeared only after one week) and were dissociated, counted and lysed in RLT Plus (Qiagen) as soon as they reached a sufficient size to be counted.

### Generation of NHP iPSCs by Sendai virus vector infection

Infection of primary cells was performed with the CytoTune-iPS 2.0 Sendai Reprogramming Kit (Thermo Fisher) at a MOI of 5 using a modified protocol. Briefly, 7 × 10^5^ urine derived cells were incubated in 100 µl of the CytoTune 2.0 SeV mixture containing three vector preparations: polycistronic Klf4–Oct3/4–Sox2, cMyc, and Klf4 for one hour at 37 °C. To control transduction efficiency 3.5 × 10^5^ cells were infected with CytoTune-EmGFP SeV. Infected cells were seeded on Geltrex (Thermo Fisher) coated 12-well-plates, routinely 10 × 10^3^ and 25 × 10^3^ cells per well. Medium was replaced with fresh Renal epithelial and mesenchymal cell proliferation medium RE/MC (ATCC) every second day. On day 5, medium was changed to mTeSR1 (Stemcell Technologies), with subsequent medium changes every second day. After single colony picking, cells were cultured in StemFit (Ajinomoto) supplemented with 100 ng/ml recombinant human basic FGF (Peprotech), 100 U/ml Penicillin and 100 μg/ml Streptomycin (Thermo Fisher).

### Immunostaining

Cells were fixed with 4% PFA, permeabilized with 0.3% Triton X-100, blocked with 5% FBS and incubated with the primary antibody diluted in 1% BSA and 0.3% Triton X-100 in PBS overnight at 4 °C. The following antibodies were used: Human alpha-Smooth Muscle Actin (R&D Systems, MAB1420), Human/Mouse alpha -Fetoprotein/AFP (R&D Systems, MAB1368), Nanog (R&D Systems, D73G4), Neuron-specific beta-III Tubulin (R&D Systems, MAB1195), Oct-4 (NEB, D7O5Z), Sox2 (NEB, 4900S), SSEA4 (NEB, 4755), EpCAM (Fisher Scientific, 22 HCLC, TRA-1-60 (Miltenyi Biotec, REA157) and the isotype controls IgG2a (Thermo Fisher, eBM2a) and IgG1 (Thermo Fisher, P3.6.2.8.1). The next day, cells were washed and incubated with the secondary antibodies for one hour at room temperature. Alexa 488 rabbit (Thermo Fisher, A-11034) and Alexa 488 mouse (Thermo Fisher, A-21042) were used in a 1/500 dilution. Nuclei were counterstained using DAPI (Sigma Aldrich) at a concentration of 1 µg/ml.

### Karyotyping

iPSCs at ~ 80% confluency were treated with 50 ng/ml colcemid (Thermo Fisher) for 2 h, harvested using TrypLE Select (Thermo Fisher) and treated with 75 mM KCL for 20 min at 37 °C. Subsequently, cells were fixed with methanol/acetic acid glacial (3:1) at − 20 °C for 30 min. After two more washes of the fixed cell suspension in methanol/acetic (3:1) we followed standard protocols for the preparation of slides with differentially stained mitotic chromosome spreads using the G-banding technique. Between 33 and 60 metaphases were analyzed per cell line.

### RT-PCR and PCR analyses

Total RNA was extracted from cells lysed with Trizol using the Direct-zol RNA Miniprep Plus Kit (Zymo Research, R2072). 1 µg of total RNA was reverse transcribed using Maxima H Minus Reverse Transcriptase (Thermo Fisher) and 5 µM random hexamer primers. Conditions were as follows: 10 min at 25 °C, 30 min at 50 °C and then 5 min at 85 °C. Quantitative polymerase chain reaction (qPCR) studies were conducted on 5 ng of reverse transcribed total RNA in duplicates using PowerUp SYBR Green master mix (Thermo Fisher) using primers specific for NANOG, OCT4, PAX6 and NESTIN. Each qPCR consisted of 2 min at 50 °C, 2 min at 95 °C followed by 40 cycles of 15 s at 95 °C, 15 s at 55 °C and 1 min at 72 °C. Cycle threshold was calculated by using default settings for the real-time sequence detection software (Thermo Fisher). For relative expression analysis the quantity of each sample was first determined using a standard curve and normalized to GAPDH and the average target gene expression (deltaCt/average target gene expression).

Genomic DNA for genotyping was extracted using DNeasy Blood and Tissue Kit (Qiagen). PCR analyses were performed using DreamTaq (Thermo Fisher). Primate primary cells were genotyped using primers that bind species-specific Alu insertions (adapted from^51^).

To confirm the transgene-free status of the iPSC lines, SeV specific primers were used described in CytoTune-iPS 2.0 Sendai Reprogramming Kit protocol (Thermo Fisher).

### In vitro differentiation

For embryoid body formation iPSCs from one confluent 6-well were collected and subsequently cultured on a sterile bacterial dish in StemFit without bFGF. During the 8 days of suspension culture, medium was changed every second day. Subsequently, cells were seeded into six gelatin coated wells of a 6-well-plate. After 8 days of attached culture, immunocytochemistry was performed using α-fetoprotein (R&D Systems, MAB1368) as endoderm, α-smooth muscle actin (R&D Systems, MAB1420) as mesoderm and β-III tubulin (R&D Systems, MAB1195) as ectoderm marker.

For directed differentiation to neural stem cells (NSCs) cells were dissociated and 9 × 10^3^ cells were plated into each well of a low attachment U-bottom 96-well-plate in 8GMK medium consisting of GMEM (Thermo Fisher), 8% KSR (Thermo Fisher), 5.5 ml 100 × NEAA (Thermo Fisher), 100 mM Sodium Pyruvate (Thermo Fisher), 50 mM 2-Mercaptoethanol (Thermo Fisher) supplemented with 500 nM A-83–01 (Sigma Aldrich), 100 nM LDN 193189 (Sigma Aldrich) and 30 µM Y27632 (biozol). Half medium change was performed at days 4, 8, 11. Neurospheres were lysed in TRI reagent (Sigma Aldrich) at day 7 and differentiation was verified using qRT PCR.

### Bulk RNA-seq library preparation

In this study two bulk RNA-seq experiments were performed, one to validate the generated iPS cells and the corresponding primary cells and one to further characterize human UDSCs derived from single colonies. For the first experiment one colony per clone corresponding to ~ 2 × 10^4^ cells and 2 × 10^3^ primary cells of each individual was lysed in RLT Plus (Qiagen) and stored at − 80 °C until processing. While for the single colony urinary cell characterization experiment we used lysate from 500 to 1000 cells per colony. The prime-seq protocol, which is based on SCRB-seq^24–26^, was used for library preparation^24–26^. The full protocol can be found on protocols.io (https://www.protocols.io/view/prime-seq-s9veh66). Even though prime-seq was used in both cases some minor differences between the two experiments exist. In particular in regards to the oligo dT primers that were used and the library preparation method as highlighted below. Briefly, proteins in the lysate were digested by Proteinase K (Ambion), RNA was cleaned up using SPRI beads (GE, 22%PEG). In order to remove isolated DNA, samples were treated with DNase I for 15 min at RT. cDNA was generated using oligo-dT primers containing well specific (sample specific) barcodes and unique molecular identifiers (UMIs). Unincorporated barcode primers were digested using Exonuclease I (New England Biolabs). cDNA was pre-amplified using KAPA HiFi HotStart polymerase (Roche) and pooled before library preparation. Sequencing libraries for the iPSC/primary cell experiment were constructed from 0.8 ng of preamplified cleaned up cDNA using the Nextera XT kit (Illumina). Sequencing libraries for the single colony experiment were constructed using NEBNext (New England Biolabs) according to the prime-seq protocol. In both cases 3′ ends were enriched with a custom P5 primer (P5NEXTPT5, IDT) and libraries were size-selected for fragments in the range of 300–800 bp.

### Sequencing

Libraries were paired-end sequenced on an Illumina HiSeq 1500 instrument. Sixteen/twenty-eight bases were sequenced with the first read to obtain cellular and molecular barcodes and 50 bases were sequenced in the second read into the cDNA fragment.

### Data processing and analysis

All raw fastq data were processed with zUMIs^52^ using STAR 2.6.0a^53^ to generate expression profiles for barcoded UMI data. All samples were mapped to the human genome (hg38). Gene annotations were obtained from Ensembl (GRCh38.84). Samples were filtered based on number of genes and UMIs detected, and genes were filtered using HTS Filter. DESeq2^54^ was used for normalization and variance stabilized transformed data was used for principal component analysis and hierarchical clustering.

Mitochondrial and rRNA reads were excluded and singleR (v1.4.0, https://bioconductor.org/packages/SingleR/) was used to classify the cells. SingleR was developed for unbiased cell type recognition of single cell RNA-seq data, however, here we applied the method to our bulk RNA seq dataset^28^. The 200 most variable genes were used in the ‘de’ option of SingleR to compare the obtained expression profiles to^55^ as well as HPCA^27^. Based on the highest pairwise correlation between query and reference, cell types of the samples were assigned based on the most similar reference cell type.

We averaged and compared pairwise expression distances for different groups (Fig. 5d): the distances among iPSC clones within and between each species (N = 14 samples), the average of the distances between 1383D2 and the urinary derived human iPSCs (N = 9) and the average of the pairwise distance between and within individuals among iPSCs and species (within individuals: N = 6 (6 individuals with more than one clone), between individuals: N = 8).

## Supplementary Information

Supplementary Information 1.

Supplementary Information 2.

Supplementary Information 3.

Supplementary Information 4.

Supplementary Information 5.

Supplementary Information 6.

Supplementary Information 7.

## Data Availability

RNA-seq data generated here are available at GEO under accession number GSE155889.

## Code availability

Code is available upon request.
