# Supplementary material for: A non-invasive method to generate induced pluripotent stem cells from primate urine
Source: Sci Rep. 2021 Feb 10;11:3516. doi: 10.1038/s41598-021-82883-0 (PMC7876031; doi:10.1038/s41598-021-82883-0)
Supplement: Supplementary file 7 — Supplementary Information 7. [file 41598_2021_82883_MOESM7_ESM.pdf]

A non-invasive method to generate induced pluripotent stem cells from primate urine

Johanna Geuder[1], Lucas E. Wange[1], Aleksandar Janjic[1], Jessica Radmer[1], Philipp Janssen[1], Johannes W. Bagnoli[1], Stefan Müller[2], Artur Kaul[3], Mari Ohnuki[1+], Wolfgang Enard[1+]

[1]Anthropology & Human Genomics, Department of Biology II, Ludwig-Maximilians-University, Großhaderner Straße 2, 82152 Martinsried, Germany

[2] Institute of Human Genetics, Munich University Hospital, Ludwig-Maximilians-University Munich, 80336 Munich, Germany

[3] Infection Biology Unit, German Primate Center, 37077 Göttingen, Germany

[+] **Corresponding author, Lead contact:**

Wolfgang Enard and Mari Ohnuki

Anthropology and Human Genomics

Department of Biology II

Ludwig-Maximilians University

Großhaderner Str. 2

82152 Martinsried, Germany

Phone: +49 (0)89 / 2180 - 74 339

Fax: +49 (0)89 / 2180 - 74 331

E-Mail: enard@bio.lmu.de, ohnuki@biologie.uni-muenchen.de

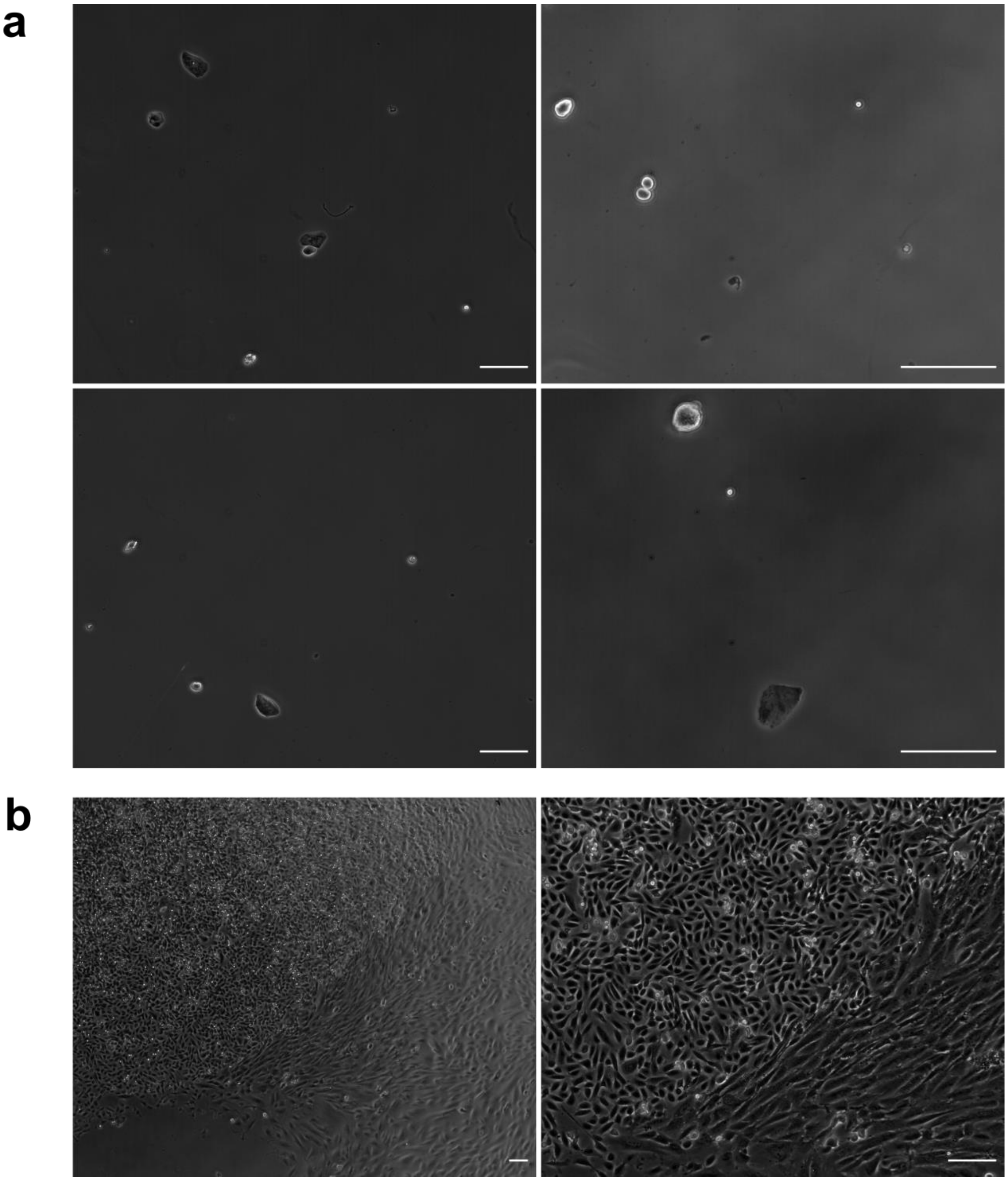

Figure S1. Cell types found in human urine samples

Different types of cells can be found in urine samples directly after collection and after proliferation. (**a**) Different cells found in human samples after centrifugation. Squamous cells as well as various smaller round cells can be found. (**b**) Two different types of cells can be distinguished after one week of culture.

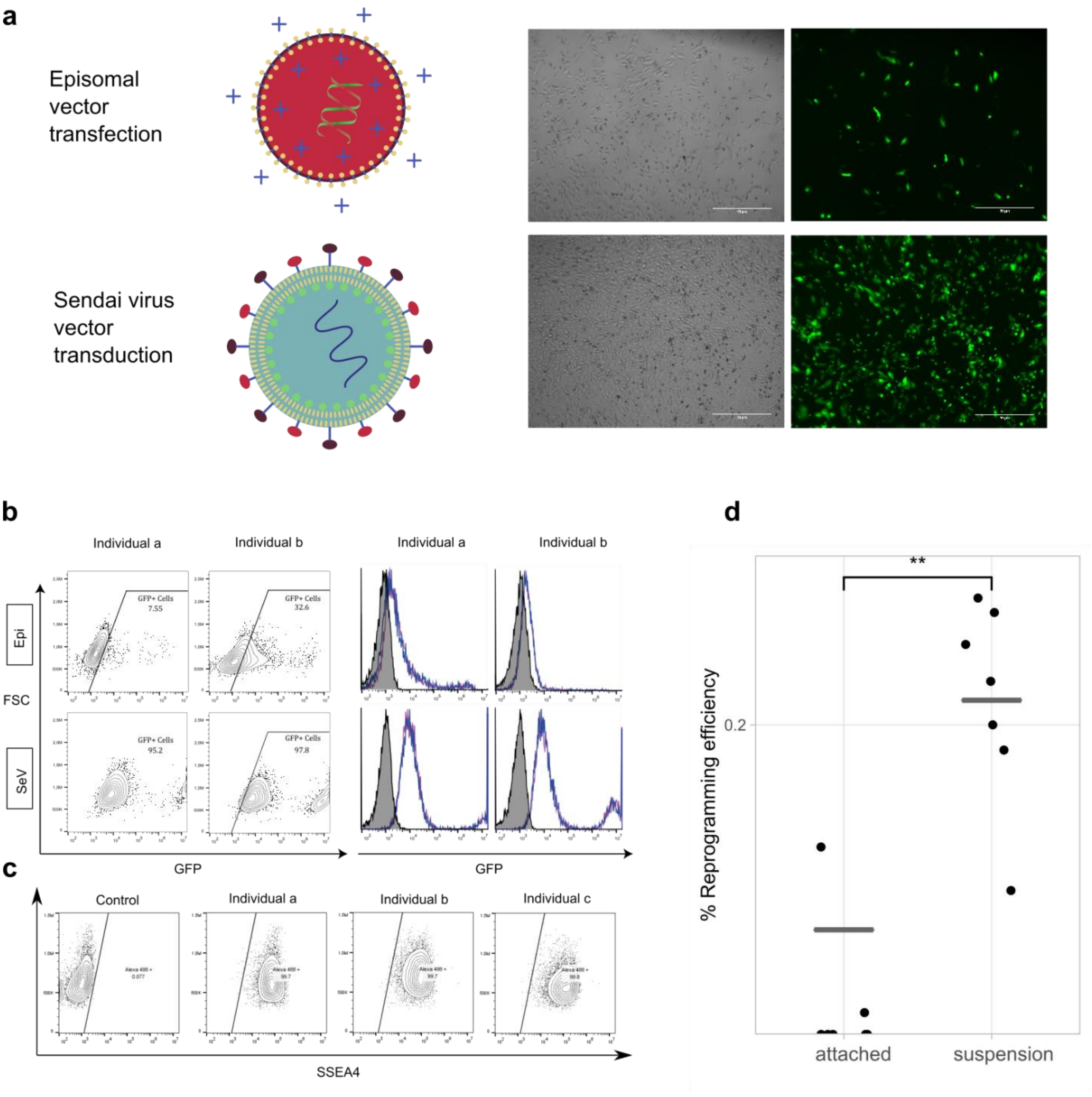

Figure S2. Transfection/Transduction efficiency of urinary cells

(a) GFP expression of urinary cells transfected with pcxle-EGFP episomal plasmids or CytoTune EmGFP transduced after 5 days **(b)** FACS analysis of GFP expressing cells 5 days post transfection/transduction **(c)** SSEA4 expression of urinary cells (**d**) Reprogramming efficiency comparison between attached and suspension reprogramming (suspension reprogramming efficiency: 0.2371%, N=7; attached reprogramming efficiency: 0.09%, N=7; Wilcoxon rank sum test: p=0.00265)

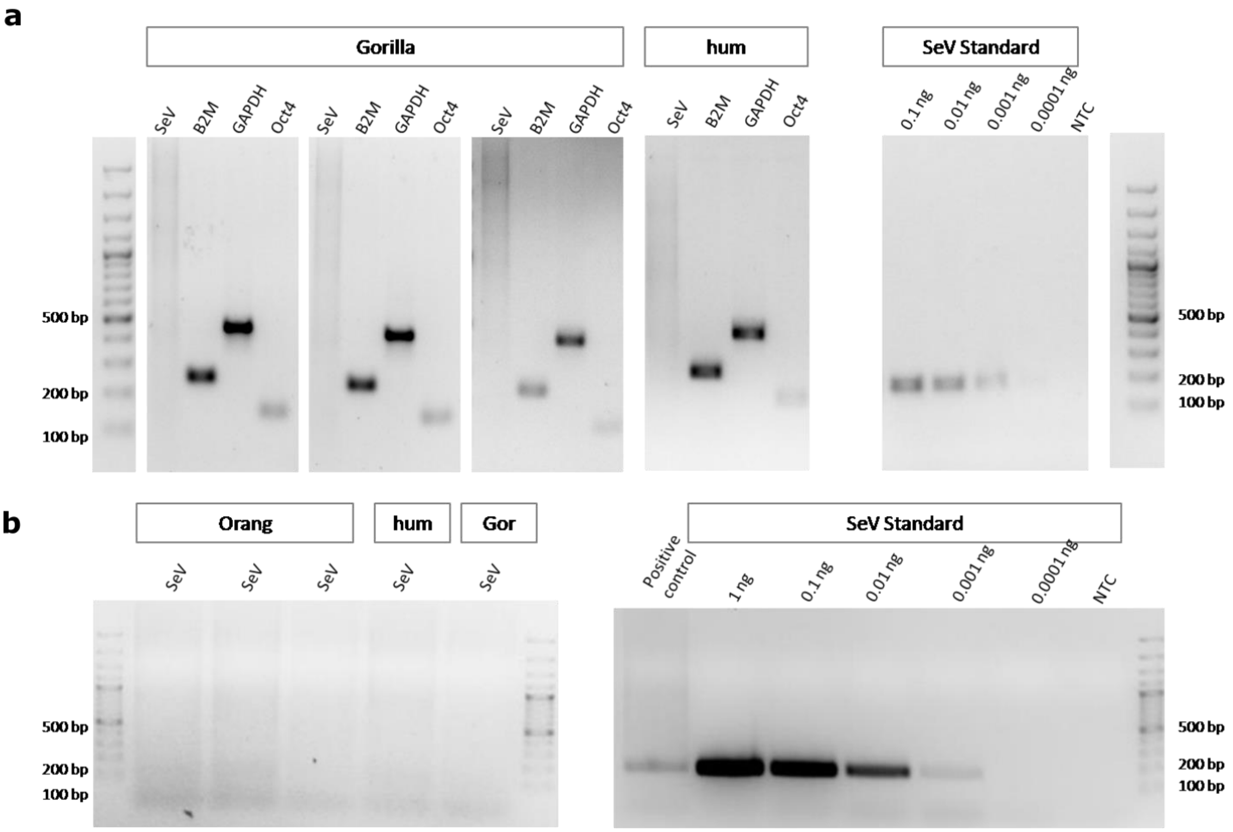

Figure S3. SeV absence verification of primate iPSC lines

Exemplary SeV absence PCR of human and nonhuman primate iPSCs. **(a)** Exemplary gorilla and human PCR targeting the SeV genome and B2M, GAPDH and OCT4 as controls. A standard dilution of the SeV product shows the sensitivity of this assay. **(b)** SEV detection PCR showing human and both primate species have no trace of SeV. The positive control are passage 1 EmGFP transduced fibroblasts.

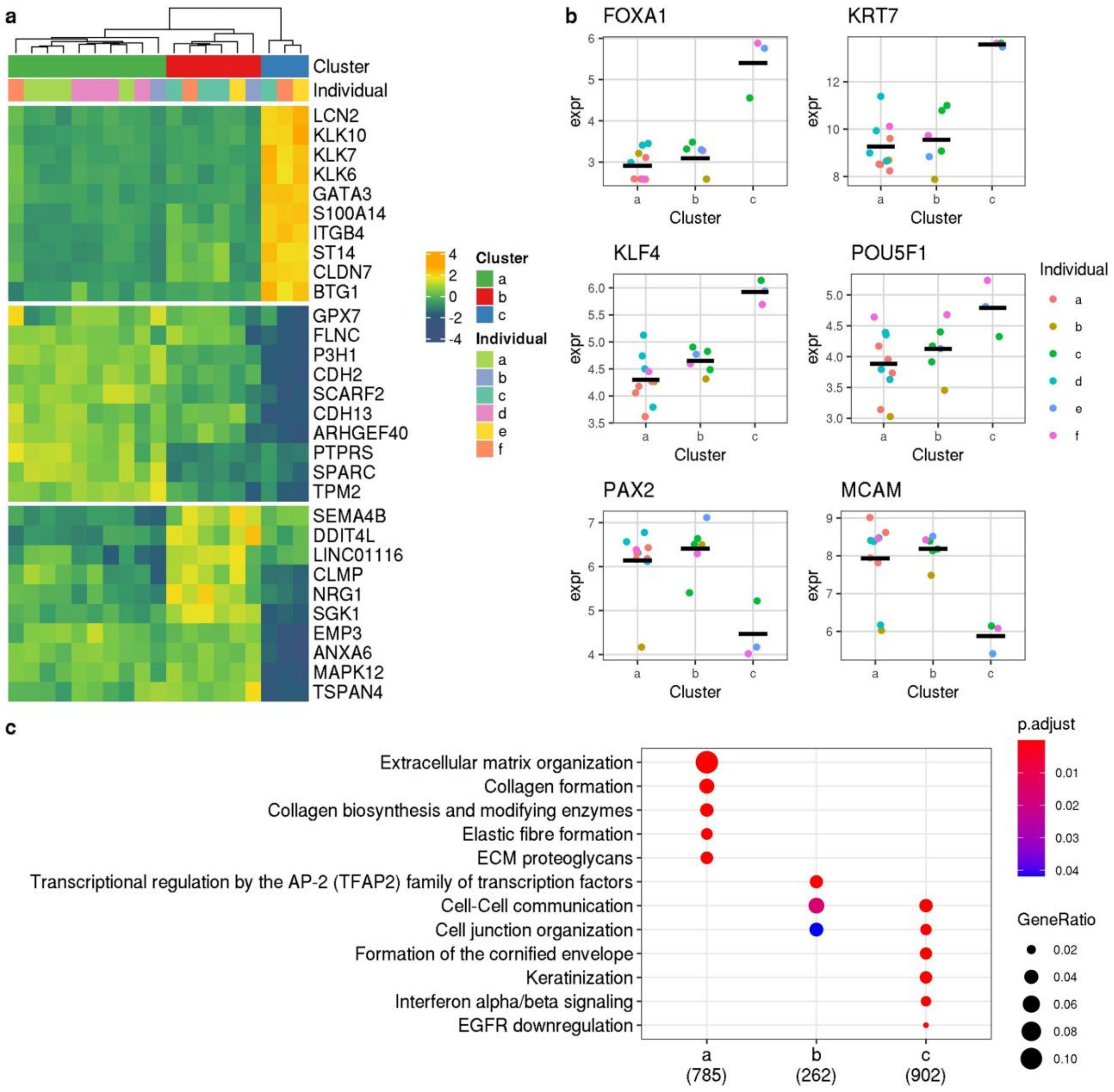

Figure S4. Characterization of human UDSCs originating from single colonies

Expression profiles of single colonies from human urine samples were subjected to further analysis. (**a**) Heatmap of top differentially expressed genes between the clusters. (**b**) Marker gene expression of different cell clusters. Cells in cluster c express urothelial cell markers (FOXA1 and KRT7). Pluripotency markers (KLF4 and POU5F1) are expressed in all clusters. PAX2 and MCAM expression is higher in cluster A and B. (**c**) Top 5 Reactome pathways enriched in the set of genes differentially expressed between one group and both other groups.

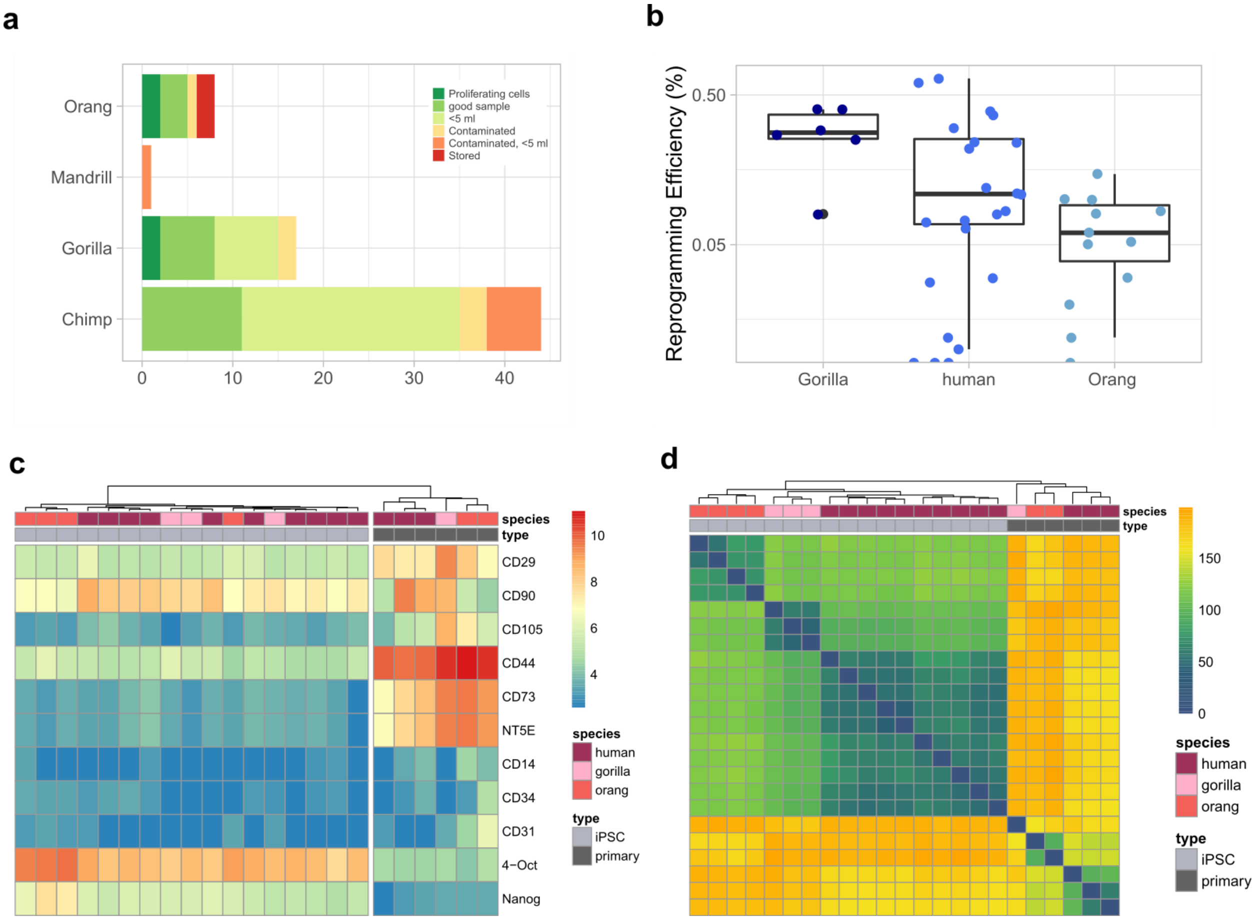

Figure S5. UDSCs and corresponding iPSC characteristics

(a) Overview of collected urine samples and properties of the samples, associated with successful isolation of proliferating cells. **(b)** Reprogramming efficiency shown as colonies per number of seeded cells between species. **(c)** Heatmap of mesenchymal stem cell and iPSC marker expression. **(d)** Euclidean distance between samples.

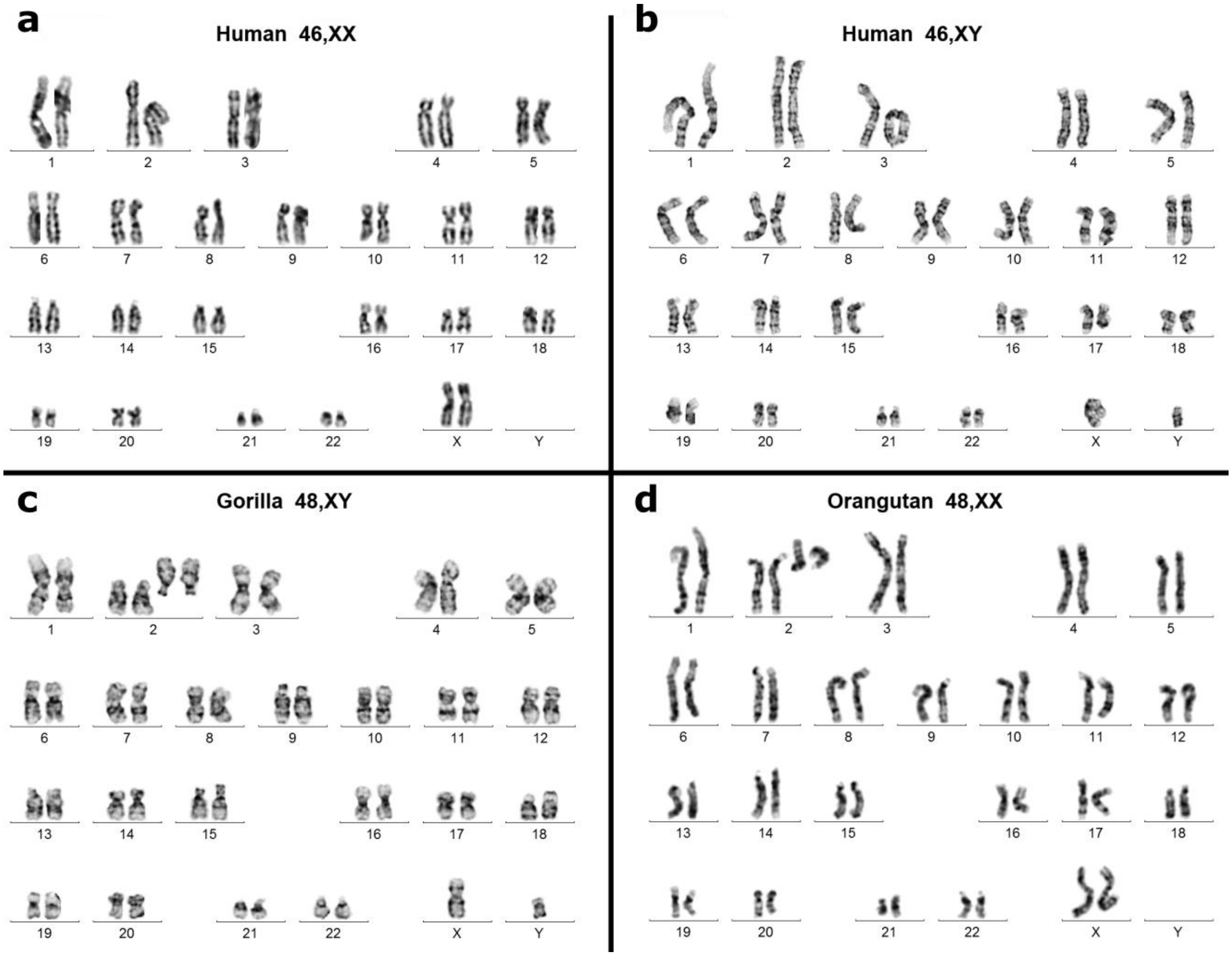

Figure S6. Karyograms of primate iPSC lines

Exemplary karyotyping analysis of human and nonhuman primate iPSCs. **(a)** human female, 46,XX **(b)** human male, 46,XY **(c)** gorilla male, 48,XY and **(d)** orangutan female, 48,XX. All karyotyped iPSC lines showed normal karyotypes without recurrent numerical or structural chromosomal alterations. Note: Ape chromosomes were ordered according to their homologies with human chromosomes and accordingly, human chromosome 2 corresponds to each two gorilla and orangutan chromosomes with homology to the long and the short arm, respectively.

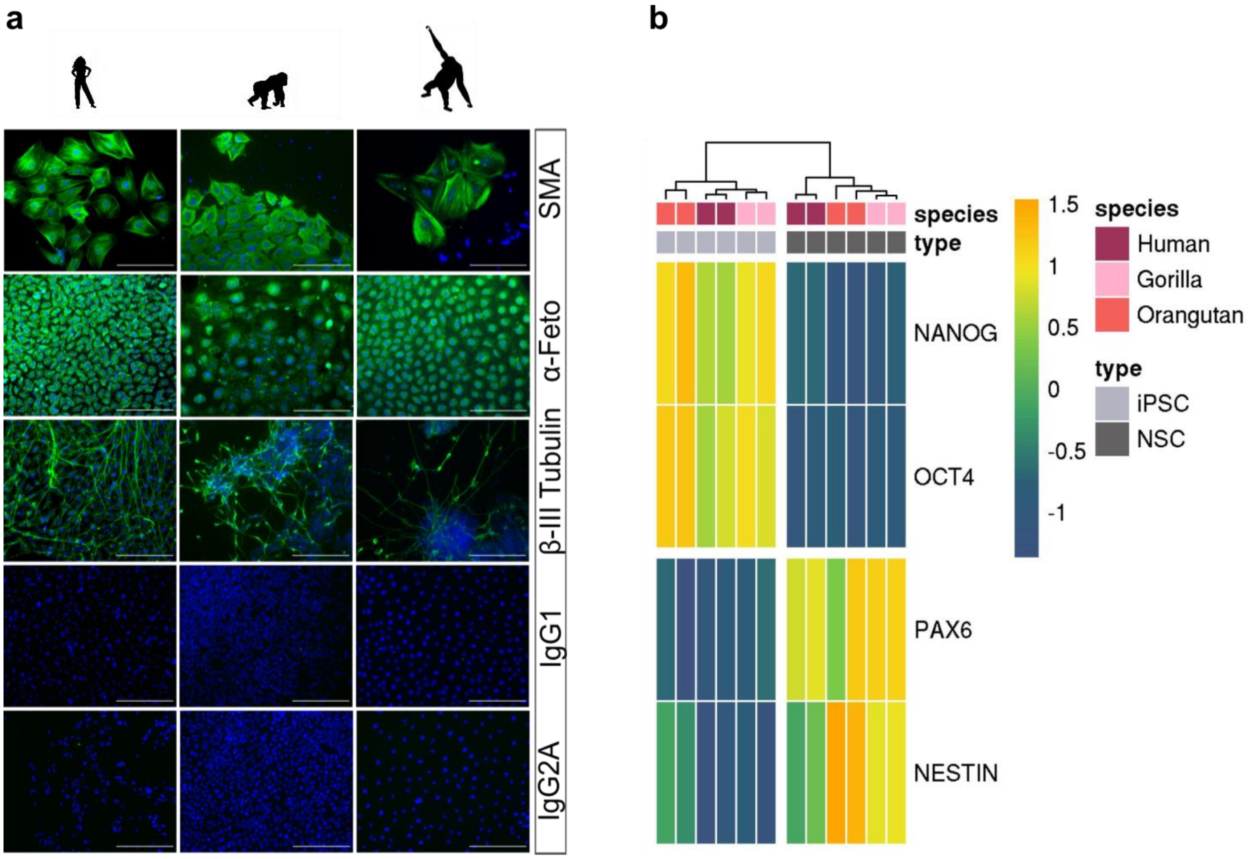

Figure S7. Differentiation capacity of iPSCs

(**a**) Immunofluorescence analyses of ectoderm (β-III Tubulin), mesoderm (α-SMA) and endoderm markers (α-Feto) after EB outgrowth. Nuclei were counterstained with DAPI. Upper 3 panels are taken from Figure 4, lower 2 panels show isotype controls for above antibodies.Nuclei are stained with DAPI in all panels; Scale bars represent 400µm. (**b**) Dual-SMAD inhibition leads to the formation of neurospheres in floating culture, confirmed by neural stem cell marker expression (NESTIN+, PAX6+) using qRT-PCR.
